# Urinary leukotriene E4 for predicting steroid sensitivity in children with nephrotic syndrome: an observational cohort study

**DOI:** 10.1007/s00467-025-06952-6

**Published:** 2025-09-30

**Authors:** Nehal Saad, Amal Osman, Mostafa Mansour, Ashraf M. Bakr

**Affiliations:** 1 Department of Pediatrics, Faculty of Medicine, Mansoura University, Mansoura, 35511 Egypt; 2 Clinical Pathology Department, Faculty of Medicine, Mansoura University, Mansoura, Egypt

**Keywords:** Biomarker, Leukotriene E4, Nephrotic syndrome, Steroid sensitive, Steroid resistant, Urinary

## Abstract

**Background:**

Nephrotic syndrome (NS) is a common pediatric kidney disorder characterized by proteinuria, hypoalbuminemia, and edema. Leukotrienes (LTs), as inflammatory mediators, may contribute to NS pathogenesis and influence treatment response. This study aimed to assess urinary leukotriene E4 (LTE4) levels in children with an initial onset of NS and evaluate their potential as biomarkers for steroid responsiveness.

**Methods:**

In this observational cohort study, 41 children with a first episode of NS and 41 age- and sex-matched healthy controls were enrolled. Patients were classified into steroid-sensitive NS (SSNS; *n* = 29) and steroid-resistant NS (SRNS; *n* = 12) groups following initial steroid therapy. Urinary LTE4 levels were measured prior to treatment, using enzyme-linked immunosorbent assay (ELISA).

**Results:**

Urinary LTE4 levels were significantly elevated in children with NS compared to controls (*p* = 0.001). Although urinary LTE4 to urinary creatinine (U cr) ratios were also higher in patients, the difference did not reach statistical significance (*p* = 0.09). No significant correlations were observed between urinary LTE4 levels and urinary protein excretion or serum albumin. Furthermore, urinary LTE4 levels did not significantly differ between SSNS and SRNS groups. A receiver operating characteristic (ROC) curve analysis showed poor predictive value of urinary LTE4 for steroid responsiveness, with area-under-the-curve (AUC) values near 0.5.

**Conclusions:**

While urinary LTE4 levels are elevated in children with NS, they failed to reliably differentiate between SSNS and SRNS. These findings suggest a limited role for urinary LTE4 as a predictive biomarker of steroid responsiveness in pediatric NS. However, future large-scale studies incorporating both plasma and urinary leukotriene profiles are warranted to validate its role in disease pathogenesis and treatment response.

**Graphical abstract:**

**Figure:**
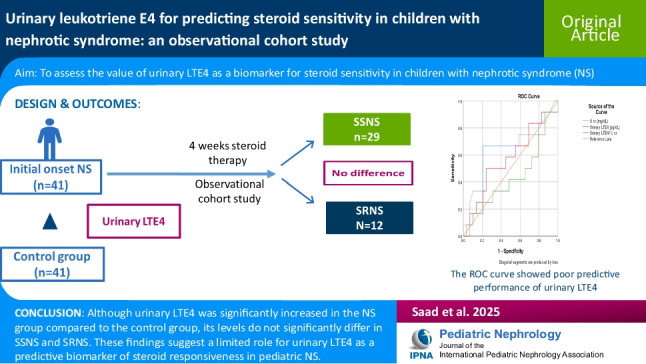
A higher resolution version of the Graphical abstract is available as Supplementary information

**Supplementary Information:**

The online version contains supplementary material available at 10.1007/s00467-025-06952-6.

## Introduction

Idiopathic nephrotic syndrome (INS) is one of the most common pediatric kidney diseases. However, its precise pathogenesis remains poorly understood. INS is classified into steroid-sensitive nephrotic syndrome (SSNS) and steroid-resistant nephrotic syndrome (SRNS) based on the clinical response to corticosteroids [1].

Corticosteroid sensitivity is observed in approximately 80–90% of patients with new-onset INS. Half of these patients go on to develop corticosteroid dependence (SDNS), while around 10–20% experience SRNS. Understanding the pathological mechanisms underlying both drug-sensitive and drug-resistant forms of INS is crucial and should precede any advances in treatment [2].

In the absence of reliable predictive biomarkers, many patients undergo prolonged courses of ineffective steroid therapy, exposing them to significant risks of toxicity and persistent disease activity [3]. The development of non-invasive biomarkers to predict steroid responsiveness could allow for earlier identification of non-responders, facilitating timely kidney biopsy and consideration of alternative immunosuppressive strategies [4].

Leukotrienes (LTs) are proinflammatory mediators derived from arachidonic acid (AA) via the 5-lipoxygenase (5-LO) pathway [5]. Previous studies have highlighted their involvement in the pathogenesis of kidney diseases, particularly in mediating immune-related kidney injury [6]. They have also been shown to significantly contribute to glomerular inflammation following both immune and non-immune insults [6, 7].

Experimental studies have demonstrated that LTs, such as LTB4, act as potent chemoattractants for neutrophils, which subsequently release proteolytic enzymes and reactive oxygen species, thereby aggravating kidney tissue injury. Moreover, leukotriene infusion in animal models has been shown to cause renal vasoconstriction, resulting in a significant reduction in kidney blood flow and glomerular filtration rate (GFR), highlighting their role in glomerular inflammation [8].

Leukotrienes are also implicated in the pathogenesis of NS. They mediate vasoconstriction of glomerular circulation, enhance the glomerular permeability to albumin, and lead to endothelial dysfunction [8, 9]. Moreover, recent studies suggest that inhibiting leukotriene production may represent a promising new therapeutic approach for patients with kidney diseases [7, 8, 10].

These findings support the biological rationale for targeting leukotriene pathways and evaluating urinary LTE4 as a non-invasive marker of kidney inflammation and potential steroid responsiveness. As the terminal metabolite of the cysteinyl leukotriene pathway, LTE4 reflects overall leukotriene activity and can reliably indicate systemic inflammatory responses. Furthermore, urinary LTE4 has been identified as the most stable and consistent biological fluid for evaluating the total production of cysteinyl-LTs [11].

To the best of our knowledge, no previous studies have evaluated urinary LTE4 as a potential biomarker for distinguishing between SSNS and SRNS. The aim of this study was to investigate its role as a non-invasive biomarker of steroid responsiveness in children with NS.

## Methods

### Study population

This is an observational cohort study comprising 41 children with NS and 41 sex- and age-matched healthy controls. All patients over 2 years of age, with an initial onset of NS, who presented to the Pediatric Nephrology Unit at Mansoura University Children’s Hospital, a tertiary center in northern Egypt, between January 2021 and January 2023, were enrolled in the study. Eligible patients were recruited consecutively to minimize selection bias. After completing 4 weeks of standard steroid therapy (oral prednisolone 60 mg/m^2^/day), patients were classified as having SSNS or SRNS according to their response, in accordance with KDIGO guidelines [12]. Of the 41 patients, 29 had SSNS and 12 had SRNS.

Included patients received only proton pump inhibitors together with standard steroid therapy, but no other routine adjunctive medications were administered at baseline. Patients with a history of gross hematuria, secondary NS, concomitant allergic diseases, a family history of kidney disease, ultrasonographic evidence of anatomical kidney abnormalities, recent acute viral illness, or other comorbidities were excluded.

The control group was recruited during routine health check-ups at our general pediatric outpatient clinic. These individuals had no clinical signs of acute infection or inflammation and had no history of chronic disease, atopy, or medication use (corticosteroids, montelukast, antihistamines) in the preceding two weeks.

### Data collection and methodology

Clinical and laboratory data were recorded for all patients, including patient history, serum albumin, urinary protein creatinine ratio (Up/Cr ratio), serum cholesterol, serum complement 3 (C3), serum creatinine, and estimated GFR (eGFR).

Urinary LTE4 levels were measured in patients and controls. In patients, urinary LTE4 was assessed at their initial presentation before starting steroid therapy. Spot urine samples were collected from all study participants. After thorough mixing, 3 mL of each sample was transferred into two separate tubes and centrifuged. The supernatant from one tube was collected and stored at – 80 °C until analysis. All samples were assayed within five months. According to previous literature, LTE4 is stable at – 80 °C for at least 5 months [13]. Urinary LTE4 concentrations were measured using a commercial ELISA kit (Catalogue No. 201–12-1552, SunRed Biotechnology Company, China), according to the manufacturer’s instructions. The intra-assay coefficient of variation (CV) was < 8%, and the inter-assay CV was < 11%, indicating acceptable assay reproducibility. The second tube was used for the measurement of urinary creatinine, following a 1:50 dilution. The creatinine concentration was then multiplied by the dilution factor to obtain the final value. Urinary creatinine (U cr) was measured using a Roche C111 analyzer. LTE4 concentrations were normalized to U cr levels and expressed as picograms of LTE4 per milligram of U cr (pg LTE4/mg creatinine).

### Outcomes

The primary objective of the study was to evaluate urinary LTE4 levels as a non-invasive biomarker to distinguish between SSNS and SRNS. The primary analysis focused on comparing urinary LTE4 levels between the SSNS and SRNS groups before starting standard steroid therapy.

### Sample size calculation

The sample size estimation was based on data from a previous study that compared urinary LTE4 levels in SSNS patients and healthy controls [14]. Using the reported means and standard deviations, we calculated a large effect size (Cohen’s *d* ≈ 3.93), suggesting that as few as three to four subjects per group would be statistically sufficient to detect a difference with > 80% power at *α* = 0.05. However, to compensate for potential dropouts or variability, we conservatively increased our target sample size, arriving at an estimated 11 participants per group.

### Statistical analysis

Data were analyzed using the Statistical Package for the Social Sciences (SPSS) program version 24 (IBM Corp., Armonk, NY, USA). Data were first tested for normality with a one-sample Kolmogorov–Smirnov test. Numbers and percentages were used to describe qualitative data. Quantitative parametric data were presented as mean (standard deviation), and quantitative non-parametric data were presented as median (range). A parametric independent *t*-test and a non-parametric Mann–Whitney test were used for comparisons between two quantitative variables. The Spearman correlation coefficient was employed to assess correlations between quantitative variables. A receiver operating characteristic (ROC) curve was used to test the diagnostic accuracy of the quantitative variable. A *p*-value less than 0.05 was considered statistically significant.

## Results

Forty-one patients with NS who met the inclusion criteria were enrolled over a 24-month period. The number of girls and boys was 18 (44%) and 23 (56%), respectively. Their mean age was 8.45 ± 3.4 years. Of those 41 patients, 12 had SRNS. Twenty-nine patients responded to steroid treatment and were labeled SSNS. The mean age of the control group was 8.06 ± 3.1 years, comprising 20 girls (49%) and 21 boys (51%). Patient demographic and laboratory data are shown in Table 1.

**Table 1:** Demographic and laboratory data of SSNS and SRNS groups

	SSNS (*n* = 29, 70.7%)	SRNS (*n* = 12, 29.3%)	*P****-***value
Age (years)	8.24 ± 3.15	8.96 ± 4.07	0.5
Sex			
Male Female	17 (58.6%) 12 (41.4%)	6 (50%) 6 (50%)	0.6
Serum albumin (g/dL)	2 (1.3–2.5)	2.0 (1.3–2.9)	01
Cholesterol (mg/dL)	366 (253–864)	358.5 (230–780)	0.9
Up/cr ratio	4 (3–4.9)	4 (3–4.5)	0.7
C3 (g/L)	1.5 (0.90–3.0)	1.3 (1.2–3.0)	0.9
Serum creatinine (mg/dL)	0.6 ± 0.1	0.58 ± 0.2	0.9
eGFR (mL/min/per 1.73 m^2^)	119 ± 19.9	127 ± 43.8	0.6

Data are expressed as median (range), mean ± SD, number (percentage). *C3*, complement 3; *eGFR*, estimated glomerular filtration rate; *SSNS*, steroid sensitive nephrotic syndrome; *SRNS*, steroid resistant nephrotic syndrome; *Up/cr*, urinary protein-creatinine ratio

**Fig. 1:**
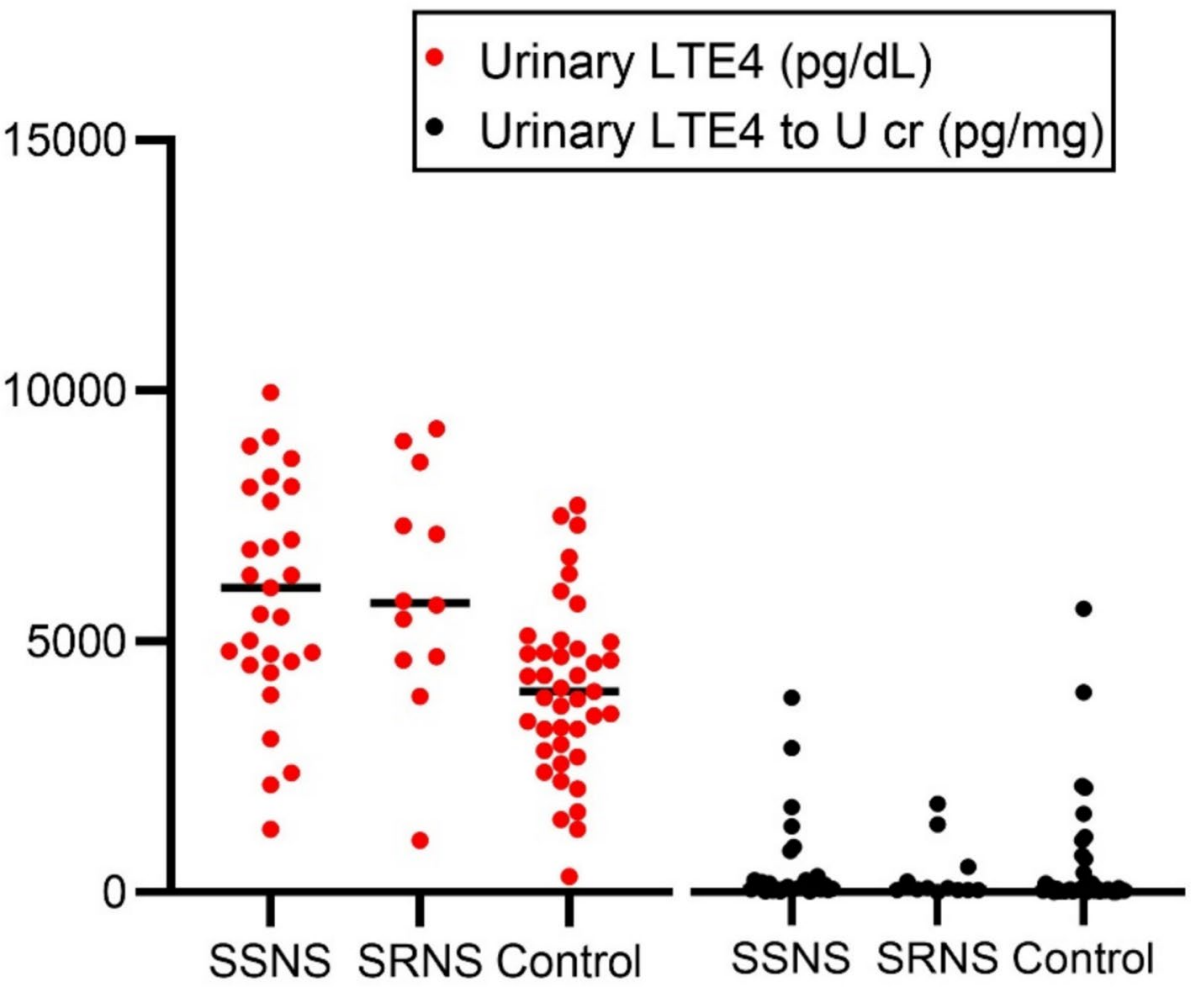
Urinary leukotriene E4 (LTE4) levels in children with steroid-sensitive nephrotic syndrome, steroid-resistant nephrotic syndrome, and healthy controls. Dot plots represent individual values of urinary LTE4 concentrations (red dots, pg/dL) and urinary LTE4 to urinary creatinine ratios (black dots, pg/mg) across the three study groups. Horizontal lines represent the median values. Statistical comparisons between each pair of groups were performed using the Mann–Whitney U test. SSNS, steroid-sensitive nephrotic syndrome; SRNS, steroid-resistant nephrotic syndrome; U cr, urinary creatinine

Urinary LTE4 was measured before the initiation of standard steroid therapy. Levels were significantly elevated in the patients with NS compared to controls (*p* = 0.001). Additionally, urinary LTE4 levels were comparable between the SSNS group (median: 5550; range: 1250–9970) and the SRNS group (median: 6480; range: 1030–9240; *p* = 0.5), but were significantly higher in both SSNS and SRNS groups compared to controls (median: 3880; range: 310–7720), with *p*-values = 0.001 for both comparisons (Fig. 1).

Although the urinary LTE4 to U cr ratio was higher in the total patient group compared to controls, the difference did not reach statistical significance (*p* = 0.09). Pairwise comparisons between subgroups also revealed no statistically significant differences (Table 2; Fig. 1).

**Table 2:** Median differences in urinary LTE4 to U cr ratio between study groups

Group	Urinary LTE4 to U cr (pg/mg)	Compared to	*P*-value	95% CI for difference
SSNS (*n* = 29)	85.1 (20.83–4080)	**SRNS**	0.5	− 586.4 to 847.7
SRNS (*n* = 12)	72.56 (6.87–1848)	**Control**	0.64	− 522.9 to 848.4
Control (*n* = 41)	66.20 (3.56–5938)	**SSNS**	0.90	− 538.9 to 474.8

Data are presented as median (range). *CI*, confidence interval; *LTE4*, leukotriene E4; *n*, number; *SSNS*, steroid sensitive nephrotic syndrome; *SRNS*, steroid resistant nephrotic syndrome; *U cr*, urinary creatinine

**Fig. 2:**
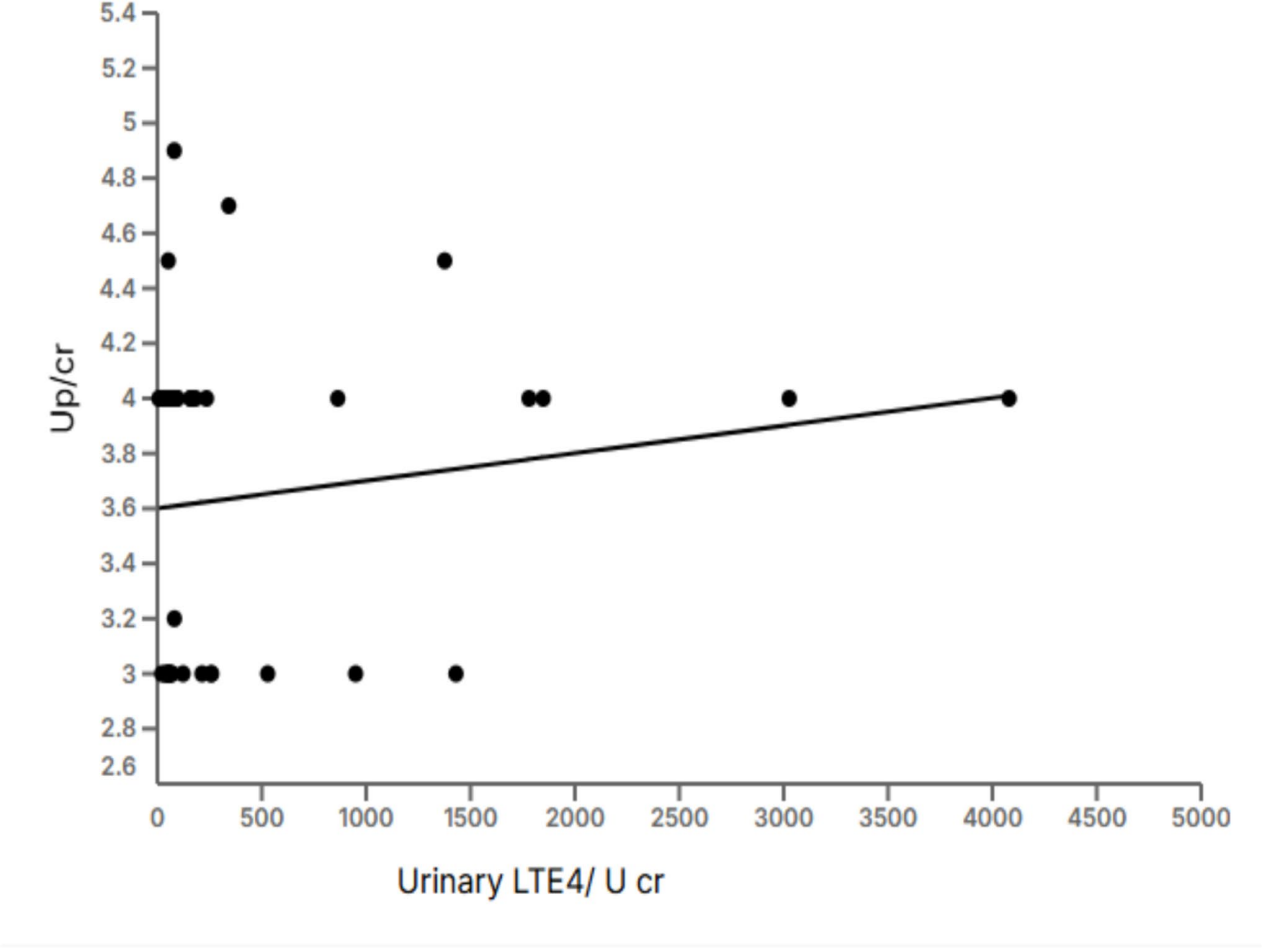
Correlation between urinary leukotriene E4 to urinary creatinine ratio (LTE4 to U cr) and urinary protein-to-creatinine ratio (Up/cr) in children with nephrotic syndrome. Each dot represents an individual participant. The *x*-axis shows urinary LTE4 normalized to urinary creatinine (pg/mg), while the *y*-axis displays the urinary protein-to-creatinine ratio (Up/cr). A linear regression line is shown. No significant correlation was observed between urinary LTE4/U cr and Up/Cr (*r* = 0.16, *p* = 0.33). U cr, urinary creatinine; Up/cr, urinary protein-to-creatinine ratio

In the present study, no significant correlation was found between urinary LTE4 to U cr ratio and either Up/cr ratio or serum albumin (*r* = 0.16, *p* = 0.33 and *r* = 0.08, *p* = 0.6, respectively) in the enrolled children with NS (Fig. 2).

**Fig. 3:**
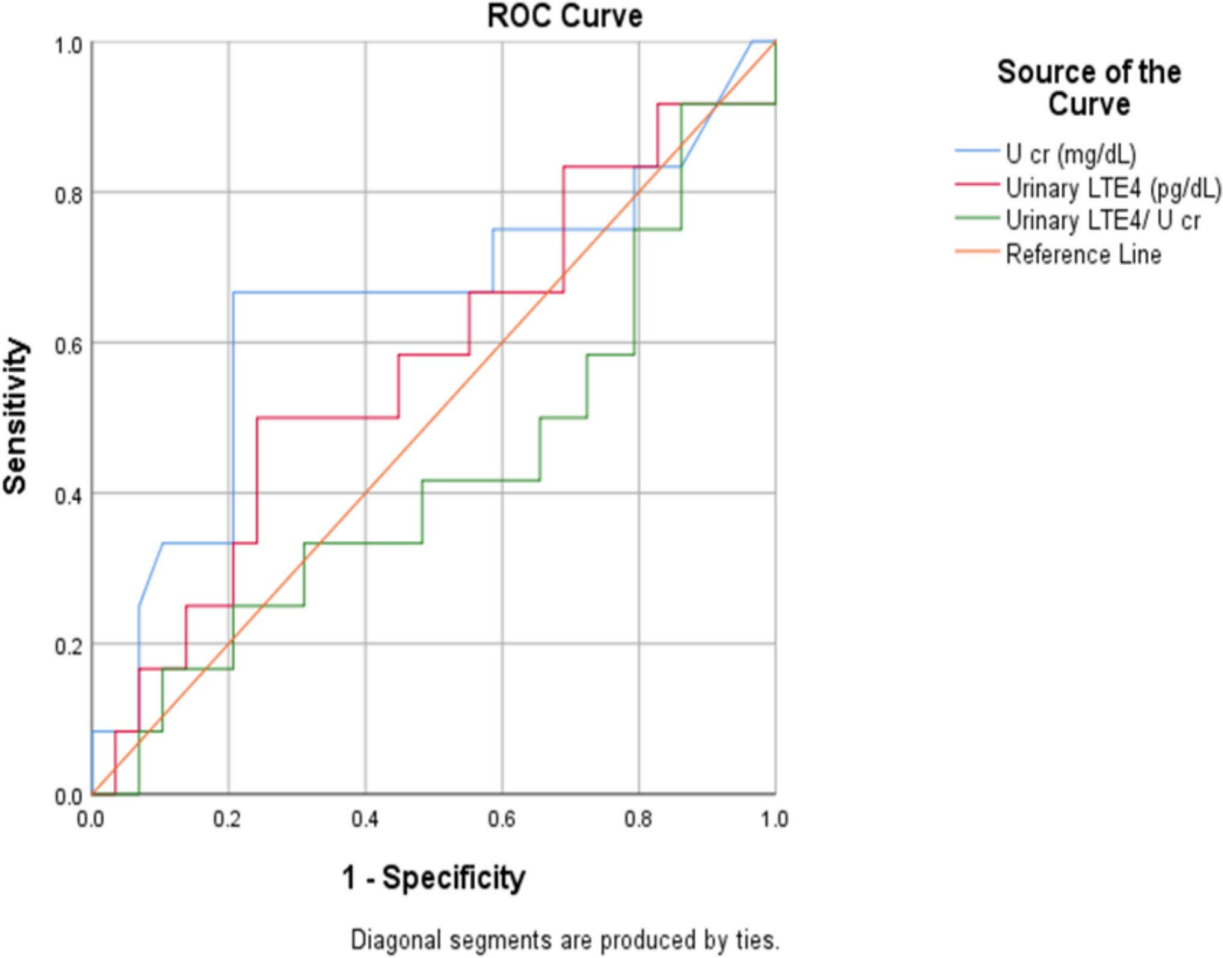
Receiver operating characteristic (ROC) curves for the prediction of steroid sensitivity in children with nephrotic syndrome using urinary leukotriene (LTE4) measures. The figure illustrates the diagnostic performance of the urinary leukotriene measures in distinguishing steroid-sensitive nephrotic syndrome (SSNS) from steroid-resistant nephrotic syndrome (SRNS). Urinary LTE4 concentration (shown in red), urinary creatinine concentration (shown in blue), and the urinary LTE4 to urinary creatinine ratio (shown in green) are shown. The orange diagonal line represents the line of no discrimination. Sensitivity is plotted against 1–specificity. Diagnostic performance was assessed by calculating the area under the curve (AUC). U cr, urinary creatinine

A ROC curve was generated to determine the sensitivity and specificity of urinary LTE4 levels and urinary LTE4 to U cr in discriminating children with steroid sensitivity. The ROC curve showed poor predictive performance of urinary LTE4 and urinary LTE4 to U cr, with an AUC approaching 0.5, suggesting little to no discriminative ability. A low combination of sensitivity and specificity was observed for urinary LTE4 to U cr with a cut-off level of 75.04 (50 and 34.5%, respectively) (Table 3; Fig. 3).

**Table 3:** Receiver operating characteristic curve for prediction of steroid sensitivity via urinary LTE4 levels

	AUC (95%CI)	*P*-value	Cut off point	Sensitivity %	Specificity %
**Urinary LTE4** (pg/dL)	0.57 (0.37–0.77)	0.47	≥ 4665	75	31
**U cr** (mg/dL)	0.64 (0.44–0.85)	0.15	≥ 83.75	66.7	79.3
**Urinary LTE4 to U cr** (pg/mg)	0.43 (0.22–0.63)	0.47	≥ 75.04	50.0	34.5

*AUC*, area under curve; *CI*, confidence interval; *U cr*, urinary creatinine; *LTE4*, leukotriene E4

## Discussion

The essential role of prednisolone and the efficacy of immunosuppressive agents in the treatment of INS significantly suggest the involvement of the immune system in its pathogenesis. Both T cells and B cells have been implicated [15]. Noninvasive biomarkers to predict steroid responsiveness could enhance prognostic accuracy, preserve kidney function, and enable a more tailored approach to management. However, there are still unmet needs [4, 16].

Previous reports have discussed the role of LTs in the pathogenesis of NS [7, 14, 17, 18]. However, no prior research has compared urinary LTE4 levels in children with SSNS and SRNS. In the current study, urinary LTE4 levels were found to be significantly elevated in children diagnosed with NS prior to the initiation of steroid therapy, in comparison to healthy controls, which could suggest a potential role of leukotriene pathways in the early inflammatory processes associated with NS. However, the urinary LTE4/U cr ratio did not differ significantly between groups, and pairwise comparisons showed no significant differences among subgroups. The wide 95% confidence intervals of median differences suggest a substantial inter-individual variability, possibly reflecting limitations in sample size. Furthermore, urinary LTE4 levels demonstrated poor predictive performance based on ROC curve analysis, limiting their utility as a biomarker in this cohort.

Although our study focused on the initial steroid response assessed at 4 weeks, as defined by the KDIGO guidelines, we acknowledge that late steroid responders represent a clinically relevant subgroup. To address this, we retrospectively reviewed the full medical records of enrolled patients over the entire two-year study period. No cases of delayed response were identified among the initially steroid-resistant patients, supporting the validity of our classification.

A prior study reported comparable findings, focusing exclusively on patients with SSNS. The urinary LTE4 to U cr ratios were observed to be markedly elevated in the SSNS group compared to the control group. Additionally, children with SSNS exhibited significantly increased levels of urinary LTB4 and LTC4 relative to the controls. In that study, plasma LTE4 and LTB4 levels were found to be significantly higher than in the controls. Yet, a significant correlation was found between proteinuria and urinary LTC4, LTD4, LTB4, and plasma LTB4 in children with SSNS, suggesting a possible involvement of LTs in the development of proteinuria and edema [14].

Consistent results were reported in another study. Patients with active NS revealed gene expression of 5-LO and LTA4 hydrolase compared to controls, whereas there was no significant difference in the degree of expression between SSNS and SRNS. In the patient group, there was also a significant positive correlation between the degree of proteinuria and the expression of 5-LO [18].

Contrary to previous findings, a prior study found no significant difference in LTB4 biosynthesis in polymorphonuclear (PMN) leukocytes between SSNS patients before steroid therapy and a control group of patients with non-inflammatory disease. Also, no significant change was noted in PMN LTB4 biosynthesis in children with SSNS after steroid therapy. The authors concluded that the inhibition of LTB4 production was not involved in the mechanism underlying steroid action in SSNS [17].

While some previous studies have reported a positive correlation between proteinuria and leukotriene levels, we did not observe such a relationship in our cohort. This discrepancy may be due to differences in sample size, or underlying patient characteristics. Our findings suggest that urinary LTE4 levels may not directly reflect proteinuria severity in all clinical contexts and could be influenced by other immunologic factors.

Despite the novel insights provided by this preliminary study, several limitations should be acknowledged. This was a single-center study with small subgroups of patients, and subgroup analyses were not considered in the initial sample size calculation. This may have contributed to the non-significant differences between SSNS and SRNS. Additionally, plasma LTE4 levels were not measured, which could have provided complementary data on systemic leukotriene activity and strengthened the interpretation of urinary findings.

In conclusion, LTs could contribute to the pathogenesis of NS. However, urinary LTE4, when measured at the time of diagnosis and prior to the initiation of corticosteroid therapy, does not appear to serve as a reliable biomarker for predicting steroid sensitivity in children with NS. Future large-scale multicenter studies incorporating both plasma and urinary leukotriene profiles are needed to better define their role in NS pathogenesis and treatment responsiveness.

## Supplementary Information

Below is the link to the electronic supplementary material.

Graphical Abstract (PPTX 132 KB)

## Data Availability

The datasets generated during and/or analyzed during the current study are available from the corresponding author on reasonable request.
