# Supplementary material for: Urinary leukotriene E4 for predicting steroid sensitivity in children with nephrotic syndrome: an observational cohort study
Source: Pediatr Nephrol. 2025 Sep 30;41(1):101–8. doi: 10.1007/s00467-025-06952-6 (PMC12686090; doi:10.1007/s00467-025-06952-6)
Supplement: Supplementary file 1 — Graphical Abstract (PPTX 132 KB) [file 467_2025_6952_MOESM1_ESM.pptx]

## Slide 1
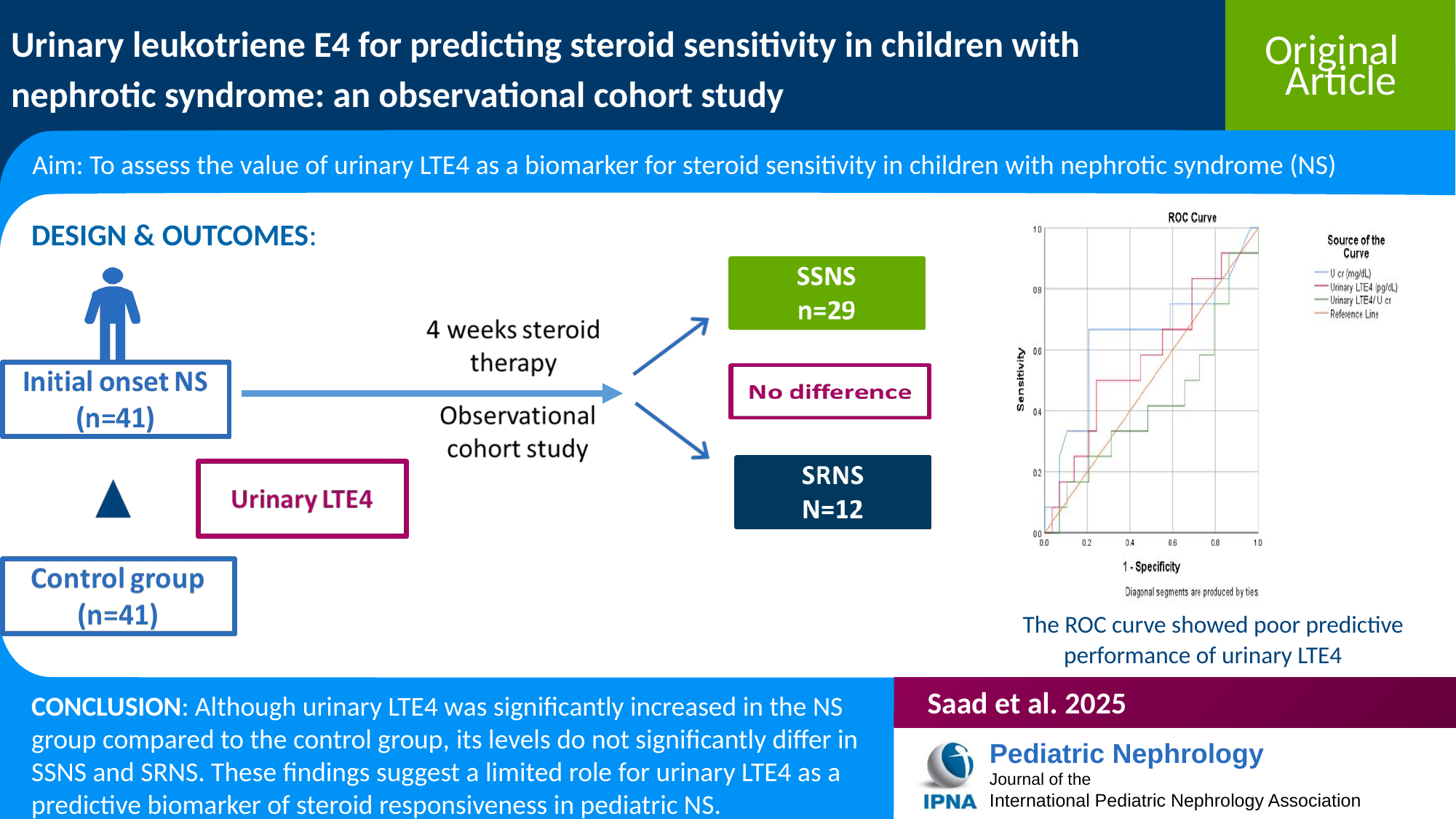

Urinary leukotriene E4 for predicting steroid sensitivity in children with nephrotic syndrome: an observational cohort study
Aim: To assess the value of urinary LTE4 as a biomarker for steroid sensitivity in children with nephrotic syndrome (NS)
DESIGN & OUTCOMES:
 The ROC curve showed poor predictive performance of urinary LTE4
Saad et al. 2025
CONCLUSION: Although urinary LTE4 was significantly increased in the NS group compared to the control group, its levels do not significantly differ in SSNS and SRNS. These findings suggest a limited role for urinary LTE4 as a predictive biomarker of steroid responsiveness in pediatric NS.
